# Neonatal Mortality in Burkina Faso: An Exploratory Analysis of Determinants and Geospatial Inequalities

**DOI:** 10.3389/ijph.2025.1608901

**Published:** 2025-11-24

**Authors:** Hervé Bassinga

**Affiliations:** Université Joseph Ki-Zerbo/Institut Supérieur des Sciences de la Population (UJKZ/ISSP), Ouagadougou, Burkina Faso

**Keywords:** neonatal mortality, determinants, inequalities, communal, SGDs

## Abstract

**Objectives:**

This article analyzes the factors associated with neonatal mortality in Burkina Faso, as well as the communal inequalities in this mortality.

**Methods:**

The analysis is based on data from the 2021 Demographic and Health Survey (DHS). It draws on a representative sample of 7,225 children. The determinants of mortality were examined using a log-binomial regression model. For the analysis of geospatial inequalities, the Richardson method was applied to classify communes according to their probability of achieving SDG target 3.2.2 by 2030, distinguishing areas with low, medium, and high likelihoods of attainment.

**Results:**

The analysis reveals an excess risk of neonatal mortality linked to male sex, multiple births, short birth intervals, low maternal education, and limited access to health services. According to the Richardson classification, all communes are on track to meet SDG target 3.2.2 (12‰ by 2030). However, 37 communes show higher residual risks requiring close monitoring.

**Conclusion:**

These results underline the importance of implementing multi-sectoral interventions adapted to territorial specificities in order to effectively maintain the reduction of neonatal mortality in Burkina Faso.

## Introduction

Every year, some 2.6 million newborns die before a month of age in the world [1]. Reducing under-five mortality, and more specifically neonatal mortality, to less than 12 deaths per 1,000 live births by 2030, is a major target of SGD n°3. Despite the progress made, the risk of death in the first month after birth remains high. According to the World Health Organization (WHO), neonatal deaths worldwide have fallen by 44% since 2000. However, in 2022, almost half (47%) of all deaths in children under 5 years of age occurred during the first 28 days of life [2]. This situation is particularly worrying in Sub-Saharan Africa, which in 2022 recorded the highest neonatal mortality rate in the world, with 27 deaths per 1,000 live births, followed by Central and Southern Asia (21‰) [2]. The main causes identified are premature birth, birth complications (neonatal asphyxia and birth trauma), neonatal infections and congenital anomalies [2]. These deaths are largely preventable, as they often result from conditions or illnesses linked to insufficient or inadequate care during childbirth, or to limited access to quality care in the first days of life.

Neonatal mortality remains a critical public health challenge in sub-Saharan Africa, shaped by a complex set of biological, maternal, and socio-economic determinants. Recent evidence highlights the role of sex of the child, multiple births, short birth intervals, maternal age and education, and limited access to skilled care as consistent predictors of neonatal survival [3–7]. Socioeconomic inequalities, particularly poverty and low maternal education, restrict care-seeking behaviors and delay access to essential newborn services [7, 8]. Beyond these individual and household-level factors, growing attention has been directed to spatial and territorial disparities, which often reflect unequal distribution of health infrastructure, security constraints, and geographic barriers to service utilization. Studies by Golding and al. [9] and Burstein et al. [10] document marked within-country heterogeneity in neonatal mortality risks, with peripheral and rural areas disproportionately affected despite overall national progress.

In Burkina Faso, in 2021, 18‰ neonatal deaths were recorded [11, 12]. This rate, although down on 1993 (43‰), remains high and well above the SDG target. Notably, neonatal mortality was relatively higher in urban areas (21‰) than in rural areas (15‰). In addition, the country has been facing a delicate security crisis for almost a decade, marked by population displacements, health center closures and disorganization of the prenatal and postnatal care system. This situation points to major spatial disparities in mortality rates, due to inequalities in access to care [13, 14].

This neonatal mortality, distinguished as early mortality when it occurs in the first 7 days of life and late mortality when it occurs between the eighth and twenty-eighth days, remains little explored in scientific work, notably due to the lack of survey data. Most studies on the determinants of infant mortality and on spatial inequalities in health have focused mainly on mortality in children under the age of five [15–22]. The few studies specifically devoted to neonatal mortality generally rely on data from health facilities [23–26], data from demographic surveillance sites, or aggregate units of analysis at regional or national levels [21, 22, 27–30].

These approaches do not provide a holistic understanding of the phenomenon, particularly geospatial inequalities on the scale of a country like Burkina Faso, and even less so at finer levels of analysis such as the commune.

This research aims to fill these gaps by analyzing the determinants and spatial inequalities of neonatal mortality in Burkina Faso using data from the latest Demographic and Health Survey (EDS 2021). The spatial scale of analysis is that of the commune, which is the smallest geographical entity with a structured administrative organization, making it possible to consider targeted actions on a territorial scale. Burkina Faso has a total of 351 communes, including 302 rural communes and 49 urban communes. The remainder of this article begins with a description of the data used and the analysis method. The second section is devoted to the analysis of the results, and the final section proposes a discussion of the main lessons, in the light of the empirical evidence highlighted in other contexts.

## Methods

### Presentation of the Study Area

Located in the heart of West Africa, Burkina Faso is a landlocked country that shares its borders with six other countries: Mali to the north and west, Niger to the east, Côte d'Ivoire, Togo, Ghana and Benin to the south. Burkina Faso inherits a dry, tropical Sudano-Sahelian climate, characterized by highly variable rainfall ranging from 350 mm in the north of the country to over 1,000 mm in the southwest. This climatic variability, more or less erratic from one geographical area to another, has an impact both on the availability of water resources for agricultural production and on children’s health. Demographically, with a high fertility rate, the country has always been characterized by a young population. The under-15 age group has consistently accounted for over 45% of the total population [12]. The fact that the population is so young places a heavy burden on the region in terms of meeting basic social needs. On the socio-economic front, enormous challenges remain. In 2019, only 29.7% of Burkina Faso’s population was literate [12]. As for the incidence of income poverty, it has not fallen significantly for decades. It fell from 44.5% in 1994 to 47% in 2009, then to 43.2% in 2021 [31].

In Burkina Faso, child mortality is falling steadily. Mortality among children under five has fallen from 187 deaths per 1,000 births in 1993 to 129 deaths per 1,000 births in 2010, and to 48 deaths per thousand in 2021 [11, 12]. As for neonatal mortality, it was eighteen (18) newborn deaths per thousand in 2021 compared with 43‰ in 1993. Although these mortality rates are steadily falling, they remain high and far from the target of 25‰ (for under-five mortality) aimed for in the SDGs by 2030. Malaria and respiratory infections are in fact the main causes of death in children under five, both in routine data from the Ministry of Health and in population observatories [32–34]. These two types of disease are responsible for more than half of all child deaths in the Ouagadougou Health and Demographic Surveillance System (HDSS) [32].

The healthcare system in Burkina Faso is organized as a three-tiered pyramid. The first level, known as the community level, comprises the Health and Social Promotion Centers (CSPS), medical centers, and medical centers with surgical units (CMA). This level serves as the main point of entry into the system and provides primary healthcare, including the management of uncomplicated deliveries, preventive and basic curative services, and health promotion activities. The second level consists of regional hospitals, which are equipped with more advanced technical facilities. These hospitals provide specialized care, act as referral centers for district-level facilities, and oversee the technical supervision of frontline structures. The third level includes national and university hospitals (CHU and CHN), located mainly in large cities such as Ouagadougou and Bobo-Dioulasso. This level manages complex and highly specialized cases and also serves as a hub for medical training and research. The pyramid is designed to ensure a progressive continuum of care, from community-based services to specialized treatment, supported by a referral and counter-referral system. However, in practice, several challenges remain, including geographic inequalities in facility distribution, a shortage of qualified health workers, and weaknesses in the referral system, which undermine efficiency, particularly in rural and peripheral areas.

In recent years, Burkina Faso has introduced several important reforms aimed at strengthening primary healthcare, expanding community-based health services, and improving access to maternal and neonatal care. Key measures include the subsidization and later full removal of user fees for deliveries and emergency obstetric and neonatal care (SONU), as well as the free healthcare policy introduced in 2016, which covers children under five and pregnant and breastfeeding women. These initiatives were designed to reduce financial barriers and promote equitable access to essential services, although significant territorial and contextual disparities persist.

**FIGURE 1:**
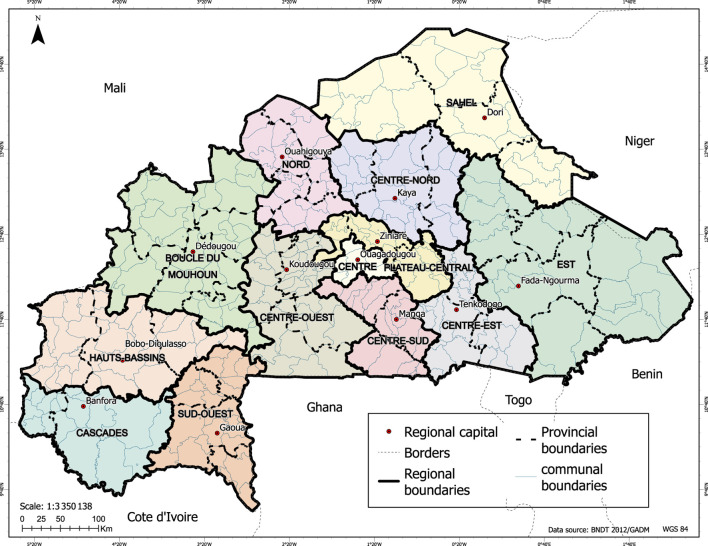
Administrative division of Burkina Faso (Burkina Faso, 2025).

Administratively, the country had a total of 13 regions, 45 provinces and 351 communes. The commune is the last and best-organized administrative entity, with local political management bodies. Figure 1 below illustrates the geographical structure of these administrative entities.

### Data Sources

The main data source used in this research is the Burkina Faso Demographic and Health Survey (DHS). It was carried out from July 30 to November 30, 2021 by National Institute of Statistics and Demography (INSD) and the DHS Program. The survey sample is nationally representative, as well as representative by region and by place of residence (urban and rural). The DHS uses a two-stage stratified sampling design. First, enumeration areas are stratified by region and urban–rural residence, then clusters are selected within each stratum using probability proportional to size. In the second stage, a fixed number of households is randomly selected from each cluster after a household listing operation. The main sampling unit is the cluster. Each region was subdivided into urban and rural components to create sampling strata, with independent sampling carried out in each stratum. In all, twenty-six strata were established. In the first sampling stage, 514 clusters were selected with probability proportional to size. In the second sampling stage, 26 households were systematically selected with equal probability from each of the clusters identified in the first stage. The survey covered a representative sample of 17,659 women of childbearing age. The collection method was a direct interview with the targets, using questionnaires implemented on CAPI.

### Target Population

Our analysis sample covers live births to women aged 15 to 49 surveyed during the DHS 2021. In all, 7,225 births were involved. These are live births to 6,627 women aged 15 to 49. These are births that took place in the last 3 years prior to the survey. A total of 137 neonatal deaths were recorded over the period.

### Analysis Variables

The variable of interest in this analysis is death of the newborn within 28 days of birth. It is coded as 1 if the newborn died before reaching 28 days of life, and 0 otherwise. In addition, the independent variables taken into account include the child’s sex, mother’s age at birth, place of delivery, birth order, intergenital interval, type of pregnancy (single or multiple), mother’s level of education, cluster distance from the nearest health and social promotion center, place of residence, region of residence, mother’s marital status and household standard of living. The measure of household living standards used in this analysis is the DHS wealth index, obtained directly from the DHS database. This variable is constructed using a principal component analysis (PCA) applied to a set of household assets and amenities (e.g., housing characteristics, access to water and sanitation, ownership of durable goods). The first component of the PCA, which captures the largest share of variance in asset ownership, is retained to generate a continuous wealth score for each household. These scores are then standardized (mean = 0, standard deviation = 1) to allow comparability across the sample. Finally, households are ranked and typically grouped into five quintiles of wealth (poorest, poorer, middle, richer, richest), which serve as a widely used proxy for socioeconomic status in demographic and health research.

### Statistical Methods

Two analytical approaches are employed in this research. First, the determinants of perinatal mortality are examined using a log-binomial regression model, which directly estimates risk ratios. To assess the validity of the model, a likelihood ratio test is performed by comparing the complete model with the null model, thereby providing an overall test of model significance. In addition, the predictive quality of the model is evaluated using the Receiver Operating Characteristic (ROC) curve, a method widely recognized as one of the most reliable tools for assessing predictive performance in regression models [35]. A model with an Area Under the Curve (AUC) greater than 70% is generally considered acceptable, reflecting a fairly good predictive ability.

The regression model is expressed as follows:

$$y_{i}∼Bernoulli\;\left(p_{i}\right)$$

$$\log\left(p_{i}\right)=\beta_{0}+\sum_{p=1}^{P}\beta_{p}x_{pi}$$ (1)

$$p_{i}=\exp\left(\beta_{0}+\sum_{p=1}^{P}\beta_{p}x_{pi}\right),0\;<p_{i}<1.$$ (2)

Where $p_{i}$ is the probability that a child dies before 28 days after birth; $x_{pi}$ are the predictor variables; the $\beta_{p}$ are the coefficients; and $\beta_{0}$ is the intercept. Under the log-binomial model, exp( $\beta_{k}$ ) is a risk ratio for a one-unit increase in $x_{k}$ (See Equations 1, 2).

In the second part, we use geospatial analysis to explore communal inequalities in neonatal mortality. This analysis is carried out in two stages.

As a first step, we assessed the spatial autocorrelation of neonatal death proportions between communes using the global Moran index, in order to verify the relevance of a spatial approach. Geospatial weighting was performed by identifying neighboring communes according to a proximity criterion: two communes are considered neighbors if they share a border (Tobler’s law). This principle is based on the law of geography formulated by Waldo Tobler, according to which “everything is connected to everything, but things that are close are more so” [36, 37]. It highlights the importance of geographical distance in structuring spatial relationships. A matrix of weights was thus constructed, attributing to each commune an influence proportional to the number of its neighbors. This weighting was used to calculate the Moran index and identify any significant spatial organization of neonatal mortality rates on a communal scale.

Secondly, to analyze spatial inequalities in neonatal mortality between communes, we used a Bayesian model with a binomial distribution, thanks to the Integrated Nested Laplace Approximation (INLA) package. This type of model makes it possible to simultaneously take into account the specific characteristics of each commune, as well as the spatial dependency effects linked to their vicinity. To this end, we have incorporated a spatial effect based on the Besag–York–Mollié (BYM) 2 model, which combines local variations with a neighborhood structure. For the model parameters, *a priori* distributions were specified. For the spatial effect, a relatively flexible configuration was chosen (parameters: 0.5 and 0.01) so as not to impose too strong an assumption from the outset. For the regression coefficients, an *a priori* distribution centered on 0 with a large uncertainty was used, corresponding to a cautious approach letting the data guide the results.

The mathematical formulation is as follows:

We consider children *i* living in communes j=1,…,351 .

The log-binomial model is specified as:

$$\log\left(p_{ij}\right)=\beta_{0}+\sum_{p=1}^{P}\beta_{p}\;x_{p,ij}+u_{j}$$

-
$\beta_{0}$ : intercept (basic risk log).
-
$x_{p,ij}$ : explanatory variables (e.g., education, standard of living, maternal status, sex of child, etc.).
-
$\beta_{p}$ : coefficients associated with covariates $x_{p,ij}$ .
-
$u_{j}$ : random effect for commune *j*, introduced to capture spatial dependency.

#### Spatial Effect Decomposition (BYM2)

In the BYM2 approach, the spatial effect is defined as:

$$u_{j}=\sqrt{1-\phi }\;v_{j}+\sqrt{\phi }\;s_{j}$$

-
$v_{j}∼N\left(0,\sigma^{2}\right)$ : unstructured effect (independent random variability between communes).
-
$s_{j}$ : spatially structured effect, defined on the basis of the neighborhood graph of the 351 communes.

$\phi \in \left[0,1\right]$ : parameter that controls the proportion of variance attributed to spatial structure.

#### Predicted Probability

The inverse of the log link gives the probability directly:

$$p_{ij}=\exp\left(\beta_{0}+\sum_{p=1}^{P}\beta_{p}\;x_{p,ij}+u_{j}\right)$$

The categorization of municipalities follows the Richardson classification, which distinguishes high, medium and low-risk areas relative to the 2030 SDG threshold for neonatal mortality (12 per 1,000 live births). For each commune *i*, we compute the posterior probability of exceeding the target, $p_{i}$ = Pr( ${NMR}_{i}$ > 12‰ | data). A commune is classified as high risk if $p_{i}$ ≥ 0.80, low risk if $p_{i}$ ≤ 0.20, and moderate risk if 0.20 < $p_{i}$ < 0.80. This approach identifies priority territories for intervention and monitoring by quantifying how likely each area is to remain above the international target.

In addition, a second categorization of communes was made using Jenks’ discretization method, applied to the *a posteriori* probability of neonatal mortality. This approach reveals finer inequalities between communes and more precisely identifies the areas most affected by this mortality.

## Results

### Descriptive Analysis of Neonatal Mortality

**FIGURE 2:**
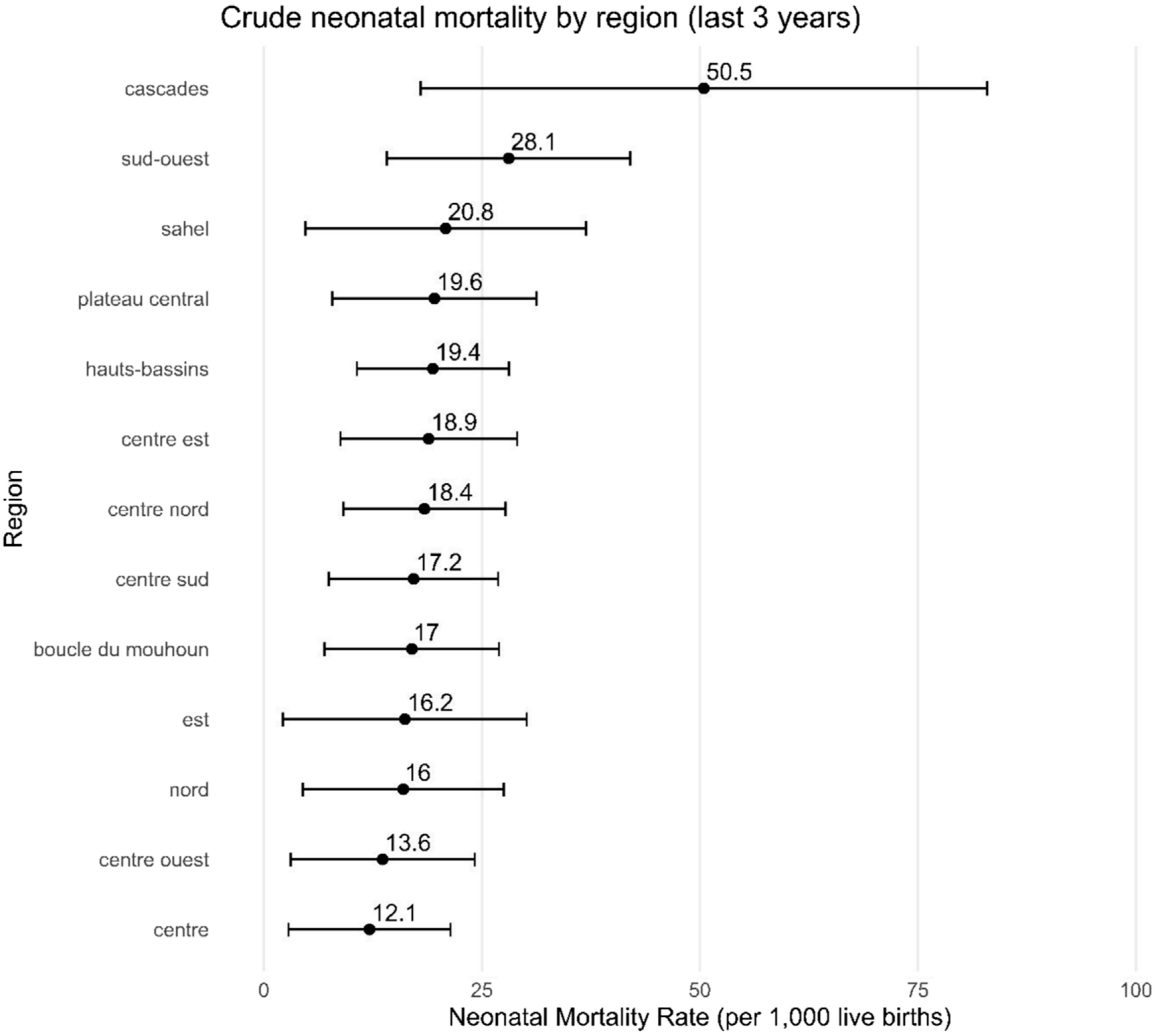
Neonatal mortality rate by region (Burkina Faso, 2021).

Before examining neonatal mortality according to individual and contextual variables, we first present the raw estimates at the regional level (cf. Figure 2). At the national level, the neonatal mortality rate is estimated at 18.05 deaths per 1,000 live births (95% CI: 14.6–21.5). However, major regional disparities are apparent, even if they are not appreciably significant. For example, while the Centre region has a neonatal mortality quotient of around 12.1 (95% CI: 2.8–21.4), the Cascades region has almost 51 (95% CI: 18.0–83.0) neonatal deaths per 1,000 live births.

Furthermore, the descriptive analysis reveals significant disparities in the occurrence of neonatal deaths, linked to the socio-economic, demographic and contextual characteristics of mothers and births (Table 1). Analysis by the length of the interbirth interval reveals marked differences (p < 0.001). Children born after an interval of less than 2 years have a neonatal mortality incidence of 5.46%, compared with 0.88% when the interval is between two and 3 years. Sex is another dimension where differences are observed: boys have a neonatal mortality incidence of 2.36%, significantly higher than that of girls (1.41%). According to the nature of the birth, the results show that multiple births have a neonatal mortality incidence of 6.37%, significantly higher than that of single births (1.69%, p < 0.001).

**TABLE 1:** Descriptive results of neonatal mortality (Burkina Faso, 2021).

Variable	*Modality*	N	%	Number of neonatal deaths	Incidence (%)	95%CI	P-value
Mother’s level of education	*No level*	4,823	66.75	87	1.8	[1.45; 2.22]	0.008
	*Primary level*	1,059	14.66	32	3.02	[2.08; 4.24]	
	*Secondary and higher*	1,343	18.59	18	1.34	[0.8; 2.11]	
Household standard of living	*Poor*	2,855	39.52	59	2.07	[1.58; 2.66]	0.452
	*Medium*	1,569	21.72	32	2.04	[1.4; 2.87]	
	*Riche*	2,801	38.77	46	1.64	[1.2; 2.18]	
Mother’s marital status	*Union*	6,902	95.53	130	1.88	[1.58; 2.23]	0.876
	*No in Union*	323	4.47	7	2.17	[0.88; 4.41]	
Birth interval	*Less than 2 years*	458	6.34	25	5.46	[3.56; 7.95]	<0.001
	*Between 2 and 3 years*	1,472	20.37	13	0.88	[0.47; 1.51]	
	*3 years and over*	3,707	51.31	66	1.78	[1.38; 2.26]	
	*First pregnancy*	1,588	21.98	33	2.08	[1.43; 2.91]	
Child’s gender	*Female*	3,544	49.05	50	1.41	[1.05; 1.86]	0.004
	*Male*	3,681	50.95	87	2.36	[1.9; 2.91]	
Place of care	*Home care or other facilities*	795	11	23	2.89	[1.84; 4.31]	0.004
	*Hospitals*	578	8	18	3.11	[1.86; 4.88]	
	*Secondary services*	5,852	81	96	1.64	[1.33; 2]	
Distance to nearest health center	*≤5 km*	431	5.97	8	1.86	[0.8; 3.62]	0.667
	*5–15 km*	2,226	30.81	47	2.11	[1.56; 2.8]	
	*>15 km*	4,568	63.22	82	1.80	[1.43; 2.22]	
Mother’s religion	*Christian*	2,109	29.19	42	1.99	[1.44; 2.68]	0.406
	*Musulmane*	4,707	65.15	84	1.78	[1.43; 2.2]	
	*Traditional*	409	5.66	11	2.69	[1.35; 4.76]	
Mother’s ethnicity	*Mossi*	4,231	58.56	64	1.51	[1.17; 1.93]	0.012
	*Autre*	876	12.12	23	2.63	[1.67; 3.91]	
	*Bobo*	864	11.96	25	2.89	[1.88; 4.24]	
	*Grue*	733	10.15	11	1.5	[0.75; 2.67]	
	*Peulh*	521	7.21	14	2.69	[1.48; 4.47]	
Type of birth	*Multiple births*	314	4.35	20	6.37	[1.4; 2.03]	<0.001
	*Unique birth*	6,911	95.65	117	1.69	[3.93; 9.67]	
Age group of mothers at birth	*<18*	380	5.26	11	2.89	[1.45; 5.12]	<0.001
	*18–34*	5,597	77.47	86	1.54	[1.23; 1.89]	
	*35–49*	1,248	17.27	40	3.21	[2.3; 4.34]	
Place of residence	*Rural*	5,112	70.75	93	1.82	[1.47; 2.22]	0.515
	*Urban*	2,113	29.25	44	2.08	[1.52; 2.79]	
Region of residence	*Centre*	696	9.63	8	1.15	[0.5; 2.25]	0.328
	*Boucle du Mouhoun*	605	8.37	11	1.82	[0.91; 3.23]	
	*Cascades*	352	4.87	12	3.41	[1.77; 5.88]	
	*Centre est*	694	9.61	14	2.02	[1.11; 3.36]	
	*Centre nord*	530	7.34	10	1.89	[0.91; 3.44]	
	*Centre ouest*	608	8.42	8	1.32	[0.57; 2.58]	
	*Centre sud*	418	5.79	8	1.91	[0.83; 3.74]	
	*Est*	461	6.38	6	1.3	[0.48; 2.81]	
	*Hauts-bassins*	763	10.56	15	1.97	[1.1; 3.22]	
	*Nord*	576	7.97	9	1.56	[0.72; 2.95]	
	*Plateau central*	655	9.07	13	1.98	[1.06; 3.37]	
	*Sahel*	291	4.03	5	1.72	[0.56; 3.96]	
	*Sud-ouest*	576	7.97	18	3.12	[1.86; 4.89]	
Means of transport	*Non-motorized*	5,801	80.29	102	1.76	[1.44; 2.13]	<0.001
	*Public transit*	35	0.48	5	14.29	[4.81; 30.26]	
	*Individual motorized*	1,389	19.22	30	2.16	[1.46; 3.07]	
Children under 5	*0*	140	1.94	31	22.14	[15.57; 29.93]	<0.001
	*1–3*	5,982	82.8	97	1.62	[1.32; 1.97]	
	*4+*	1,103	15.27	9	0.82	[0.37; 1.54]	
Global		7,225	100	137	1.90	[1.59; 2.24]	

With regard to the mother’s age at delivery, children born to mothers aged 35 to 49 have a neonatal mortality incidence of 3.21%, higher than that observed among children born to younger mothers (18–34 years), with a statistically significant difference (p < 0.001).

Differences were also observed according to ethnicity. Children born to mothers belonging to the Bobo (2.89%) and Peulh (2.69%) groups had higher neonatal mortality rates than those of other ethnic groups (p < 0.001). Likewise, the results relating to the means of transport used to reach the place of delivery indicate a marked disparity: the use of non-conventional means or public transport is associated with very high incidences of neonatal mortality, reaching 14.29%, which reflects significant difficulties in accessing obstetric services.

Finally, the presence of a child under five living in the household significantly influences the incidence of neonatal mortality. Indeed, the incidence of neonatal mortality is 22.14% when there is at least one child under 1 year in the household.

### Multivariate Analysis of Neonatal Mortality Outcomes

The set of variables included in the analysis model gives an AUC of 0.81 (as shown by the ROC curve in Supplementary Figure S1), indicating good discriminating ability and providing non-random predictions. Thus, the holistic analysis of neonatal mortality highlights a set of sociodemographic, biological and contextual factors significantly associated with an increased probability of death within the first 28 days of life (See Table 2).

**TABLE 2:** Results of log-binomial analysis of factors associated with neonatal mortality (Burkina Faso, 2021).

Variables	RR	95% IC	P-value
Mother’s level of education			
*Secondary level and above*	*Ref*	*Ref*	*Ref*
*Primary level*	2.27	1.29, 4.01	0.005
*No level*	1.38	0.79, 2.39	0.3
Household standard of living			
*Rich*	*Ref*	*Ref*	*Ref*
*Middle*	1.30	0.81, 2.08	0.3
*Poor*	1.31	0.80, 2.15	0.3
Mother’s marital status			
*Union*	*Ref*	*Ref*	*Ref*
*Not in Union*	0.72	0.33, 1.54	0.4
Birth interval			
*Less than 2 years*	*Ref*	*Ref*	*Ref*
*Between 2 and 3 years*	0.19	0.10, 0.37	<0.001
*3 years and over*	0.28	0.18, 0.45	<0.001
*First pregnancy*	0.46	0.24, 0.89	0.021
Child’s gender			
*Female*	*Ref*	*Ref*	*Ref*
*Male*	1.60	1.14, 2.24	0.007
Birth order	1.08	0.97, 1.21	0.2
Place of care in the event of illness			
*Home care and other facilities*	*Ref*	*Ref*	*Ref*
*Hospitals*	0.96	0.52, 1.78	>0.9
*Secondary services*	0.56	0.36, 0.88	0.011
Distance to nearest health center			
*Less than 5 km*	Ref	Ref	Ref
*Between 5 and 15 km*	1.01	0.59, 1.74	>0.9
*More than 15 km*	0.73	0.50, 1.05	0.092
Religion			
*Christian*	*Ref*	*Ref*	*Ref*
*Muslim*	0.84	0.55, 1.27	0.4
*Traditional*	1.15	0.60, 2.22	0.7
Mother’s ethnicity			
*Mossi*	*Ref*	*Ref*	*Ref*
*Autre*	1.32	0.74, 2.36	0.3
*Bobo*	1.27	0.69, 2.34	0.4
*Gure*	1.29	0.57, 2.94	0.5
*Peulh*	2.11	1.07, 4.14	0.031
Type of birth			
*Single birth*	*Ref*	*Ref*	*Ref*
*Multiple birth*	3.63	2.35, 5.61	<0.001
Age of mothers at birth			
*<18*	*Ref*	*Ref*	*Ref*
*18–34*	0.88	0.44, 1.75	0.7
*35–49*	1.41	0.61, 3.27	0.4
Place of residence			
*Rural*	*Ref*	*Ref*	*Ref*
*Urban*	1.14	0.75, 1.73	0.5
Region of residence			
*Centre*	*Ref*	*Ref*	*Ref*
*Boucle du Mouhoun*	2.36	0.92, 6.08	0.076
*Cascades*	3.87	1.40, 10.7	0.009
*Centre est*	2.36	0.99, 5.62	0.053
*Centre nord*	1.85	0.75, 4.57	0.2
*Centre ouest*	1.68	0.59, 4.81	0.3
*Centre sud*	2.16	0.78, 5.99	0.14
*Est*	1.63	0.47, 5.61	0.4
*Hauts-bassins*	2.14	0.86, 5.32	0.10
*Nord*	1.84	0.69, 4.92	0.2
*Plateau central*	2.67	1.08, 6.58	0.033
*Sahel*	1.19	0.34, 4.13	0.8
*Sud-ouest*	2.38	0.93, 6.11	0.071
Means of transport			
*Non-motorized*	*Ref*	*Ref*	*Ref*
*Public transit*	8.57	3.52, 20.9	<0.001
*Motorized individual*	1.52	0.99, 2.33	0.055
Children under 5			
*0*	*Ref*	*Ref*	*Ref*
*1–3*	0.08	0.06, 0.12	<0.001
*4+*	0.04	0.02, 0.08	<0.001

Abbreviations: CI, confidence interval; RR, relative risk.

Among child characteristics, male sex stands out as a factor strongly associated with excess neonatal mortality. Compared to girls, boys show a significant excess risk (RR = 1.60; p = 0.007), confirming a male biological vulnerability during this critical period. The type of birth is also an important differentiating factor. Children from multiple births are almost three times more likely to die within the first 28 days of life than those from single births (RR = 3.63; p < 0.001).

With regard to ethnicity, children born to mothers belonging to Peulh groups were 2.11 times more likely to die during the first 28 days of life, compared with those born to mothers belonging to Mossi groups (p = 0.031), reflecting possible cultural, territorial or healthcare access disparities. The inter-genetic interval has a strong protective effect on neonatal mortality. Compared with children born less than 2 years after a previous birth, those born after a birth interval of 3 years or more have a 72% lower risk of dying within the first 28 days of life (p < 0.001). The risk is also by 81% (p < 0.001) among children born after an interval of two to 3 years, and by 54% (p < 0.021) among those from a first pregnancy.

With regard to mother-related factors, the mother’s level of education also plays a role in neonatal mortality. Children born to mothers with primary education are 2,27 more likely to die within the first 28 days of life, compared with children born to mothers with secondary education and above (p = 0.005).

Geographically, some regions have significantly higher mortality risk levels. This is particularly true of the Cascades (RR = 3.87; p = 0.009), Centre-Est (2.36; p = 0.053) and Central Plateau (RR = 2.67; p = 0.033) regions, where children have a significantly higher probability of death than in other regions. Access to care emerges as a critical dimension. Children who seek care in secondary health centers are 44% (p = 0.011) less likely to die before their 28th birthday than those who seek care at home or elsewhere than in a health center. In addition, the means of transport used to access care had a marked influence on newborn survival. The use of public transport is associated with an extremely high risk of neonatal mortality (RR = 8.57; p < 0.001).

### Analysis of Communal Disparities in Neonatal Mortality Risk Using Richardson’s Approach

The estimate of Moran’s I at the commune scale yields I = −0.0128 (p = 0.561), indicating that there is no significant spatial autocorrelation, which means that neonatal deaths are randomly distributed across communes. In addition, estimating the probability for each commune of achieving a neonatal mortality level less than or equal to the 12‰ threshold by 2030 under SDG 3.2.2 revealed that all communes in Burkina Faso have a low level of risk (predicted probability <0.80). This means that the 351 communes of Burkina Faso will meet SDG 3.2.2, all else being equal. However, 34 communes fall within a range that warrants vigilance (Cf. Supplementary Table S2). All of these communes have probabilities between 0.20 and 0.80, which represents a moderate risk. These include, among others, Kpuéré, Madouba, Legmoin, Boussou-Koula, Niankôrôdougou, Botou, and Batié (Urban commune).

**FIGURE 3:**
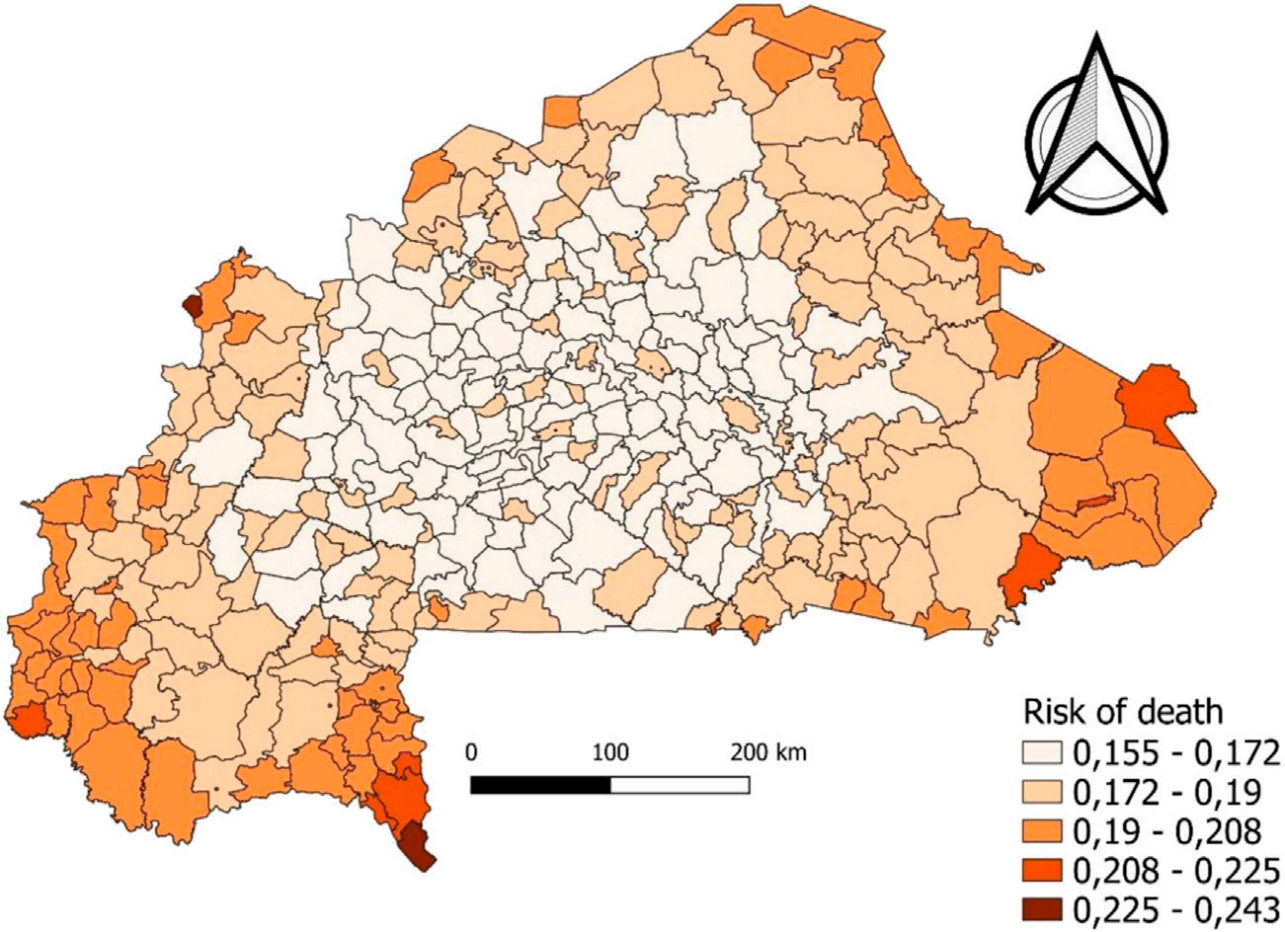
Mapping of neonatal mortality, cut according to Jenks discretization (Burkina Faso, 2021).

In addition, analysis of relative risk predictions at the commune level, based on Jenks’ discretization, reveals spatial inequalities with a dual center–periphery structure, where neonatal mortality is much higher the farther one moves away from the center of the country, a low-risk zone relative to the country’s peripheries (cf. Figure 3). Peripheral communes such as those mentioned above should be monitored closely. In contrast, the communes of Ziniaré, Nanoro, Ténado, and Boken, among others, present a relatively low risk of neonatal deaths.

## Discussion

The results of this study reveal the multidimensional complexity of neonatal mortality in Burkina Faso, characterized by the interaction of biological, sociodemographic, geographical and structural factors. This complexity manifests itself both in individual differences and in the territorial disparities highlighted by our statistical and spatial analyses.

The influence of the mother’s level of education observed in this study confirms the findings of previous studies, notably those by Lankoandé et al. in Ouagadougou [32] and Alam et al. in India, and by Bora and Saikia [28] in Bangladesh, which showed that a low level of education is a factor limiting access to health information, prenatal care and adequate management. In our study, this association remains marked, particularly among mothers with primary education.

Biologically and clinically, the results confirm the particular vulnerability of male newborns. This vulnerability can be explained by lung immaturity, greater susceptibility to infections, and less advanced immune development at birth [14, 34]. Furthermore, the high risk of mortality in multiple-birth infants observed in this study is consistent with the observations of Nagalo and colleagues and Ouédraogo and colleagues [24, 38], who identify prematurity, neonatal asphyxia and low birth mass as the main clinical challenges in these cases.

Cultural and community dimensions, which are often underestimated, also play a decisive role. Ethnicity, particularly in the Peulh group, is associated with a high risk, corroborating the findings of Cau and co-authors [19], which highlight the socio-cultural obstacles to access to care. These disparities may be explained by differences in risk perception, social norms or relationships with biomedical structures.

Interestingly, the mother’s marital status also appears to influence neonatal mortality. In our study, children born to unmarried mothers had a lower risk of neonatal death, a finding also noted in India by Ram and co-authors [26], where greater decision-making autonomy for unmarried women can translate, in some contexts, into more rapid and direct recourse to care.

These findings are part of an overall context of gradual but uneven decline in infant mortality. Despite real progress [2, 37], profound structural and territorial inequalities are holding back the achievement of our objectives. In Burkina Faso, although progress has been made, the situation remains worrying, particularly in rural areas, as UNICEF [1] points out.

Moreover, factors such as region of residence confirm territorial vulnerability patterns that are well established in the literature. This consistency between empirical findings and prior research highlights the need to account for regional disparities in the planning of neonatal health policies. The regional inequalities observed, particularly in the Cascades, Plateau Central, Centre-Est (RR = 2.36, p = 0.053) and Sud-Ouest(RR = 2.38, p = 0.071) regions, confirm well-known structural geographical disparities [32, 36], and already pointed out in the RGPH 2019 reports [39]. Indeed, peripheral, underserved areas with a high level of health vulnerability confirm the idea of a more refined territorialization of healthcare policies, adapted to local contexts [30, 35]. Conditions of geographical accessibility to healthcare are emerging as a critical issue. The use of public transport or non-motorized means of transport is strongly associated with excess neonatal mortality, revealing the urgent need to improve medical evacuation arrangements. These results are consistent with the findings of Niang [35] on non-use of healthcare in rural areas, and of Johnson and co-authors [36], which call for a rethinking of primary healthcare based on territorial equity.

Spatial autocorrelation analysis using Moran’s I did not confirm the presence of significant autocorrelation in neonatal mortality across the country’s 351 communes. This result suggests that neonatal mortality is randomly distributed in space and does not tend to cluster in specific geographic areas.

However, the absence of global autocorrelation (non-significant Moran’s I) does not rule out the existence of local descriptive contrasts. This result should be interpreted with caution and, if necessary, confirmed by local indicators such as the Local Indicators of Spatial Association (LISA) [40].

An in-depth Bayesian analysis enabled us to better characterize this dynamic with a view to identifying high-risk areas, where the probabilities of excess risk exceed 0.8 according to the Richardson classification [41]. This exploration suggests that most communes in Burkina Faso are on track to meet SDG 3.2.2, which seems rather optimistic given the deleterious security situation that continues to affect the local supply of care. It is evident that between the analysis period (3 years back from 2021) and 2030, this trajectory is strewn with obstacles and could hold surprises for many communes.

The spatial mapping analysis reveals marked spatial inequalities in the *a posteriori* probability of neonatal mortality, characterized by a center–periphery gradient. The eastern, far northern, and far western regions of the country are the most affected, in contrast with the central zone, which appears relatively protected. This spatial pattern likely reflects the combined effect of several structural determinants: unequal distribution of health infrastructure, differential exposure to insecurity, and geographic barriers that increase both the cost and the time required to access health services. A dual center–periphery structure of mortality has already been demonstrated in Burkina Faso in a study of children under five [41].

These findings are consistent with the analyses of Dwyer-Lindgren et al. [20] and Rican and Vaillant [42], who highlight the importance of territorializing health policies in order to effectively target the most vulnerable areas. They also corroborate the results of the 2019 Population and Housing Census (RGPH), which showed that several rural peripheral areas remain left behind despite national efforts to improve maternal and child health [39].

### Limitation and Strength of the Study

This study has a number of strong points that reinforce the reliability of the results obtained to inform public policy. Firstly, it is based on recent, representative and disaggregated national data from the EDS 2021, enabling a detailed analysis at the scale of Burkina Faso’s 351 communes. In addition, the joint use of multivariate statistical models and spatial Bayesian approaches provides a sound methodology for identifying determinants and highlighting spatial inequalities in neonatal mortality.

However, certain limitations need to be highlighted. Indeed, the analysis is based on declarative data, susceptible to recall bias, and on a cross-sectional approach, limiting the causal interpretation of the associations observed. In addition, certain relevant contextual variables, such as the actual quality of care or local conflict dynamics, are not directly integrated. A further limitation relates to the security situation during data collection, which led to incomplete coverage in some regions, including the Sahel, East, Centre-North and Boucle du Mouhoun. This may have contributed to an underestimation of neonatal deaths in certain communes, such as Djibo, where the estimated risk appears unexpectedly low compared to known regional trends.

Despite these limitations, the results obtained remain sufficiently robust and relevant to support the implementation of targeted, territorialized and evidence-based strategies within the framework of neonatal health policies.

### Conclusion

The results of this study show that neonatal mortality in Burkina Faso remains a major public health challenge, shaped by strong biological, social, and territorial disparities. While individual factors such as the child’s sex, birth interval, and type of delivery significantly influence the risk of death, structural inequalities related to access to care, socio-economic status, and socio-cultural barriers play an equally decisive role. Spatial analysis revealed a core of priority communes with particularly high risks including both rural and urban areas challenging the assumption that urban environments systematically guarantee better health outcomes. Moreover, the Jenks discretization highlights that medium-risk communes are often concentrated around high-risk ones, pointing to a territorial contiguity of vulnerabilities.

In this context, achieving the Sustainable Development Goals (SDGs) for reducing neonatal mortality requires the implementation of multi-sectoral, territorially differentiated strategies. These must be informed by disaggregated, spatially explicit data, and adapted to the realities of both rural peripheries and underserved urban zones. Only a contextualized and equitable approach, grounded in scientific evidence and sensitive to local specificities, will make it possible to achieve a sustained reduction in neonatal mortality in Burkina Faso [20, 34, 43–45].
