# Supplementary material for: Neonatal Mortality in Burkina Faso: An Exploratory Analysis of Determinants and Geospatial Inequalities
Source: Int J Public Health. 2025 Nov 24;70:1608901. doi: 10.3389/ijph.2025.1608901 (PMC12682704; doi:10.3389/ijph.2025.1608901)
Supplement: Supplementary file 1 [file DataSheet1.pdf]

SUPPLEMENTARY FILES

Table 1: Neonatal mortality rate by region and at national level

Region	Neonatal mortality rate	Lower bound	Upper bound
**National**	18.05	14.56	21.54
**Boucle Du Mouhoun**	16.97	6.97	26.98
**Cascades**	50.47	17.97	82.96
**Centre**	12.12	2.81	21.42
**Centre Est**	18.91	8.77	29.04
**Centre Nord**	18.42	9.11	27.73
**Centre Ouest**	13.62	3.06	24.18
**Centre Sud**	17.17	7.47	26.87
**Est**	16.17	2.19	30.15
**Hauts-Bassins**	19.39	10.66	28.12
**Nord**	15.99	4.46	27.52
**Plateau Central**	19.56	7.86	31.26
**Sahel**	20.84	4.74	36.94
**Sud-Ouest**	28.07	14.11	42.02

Figure 1 : ROC curve

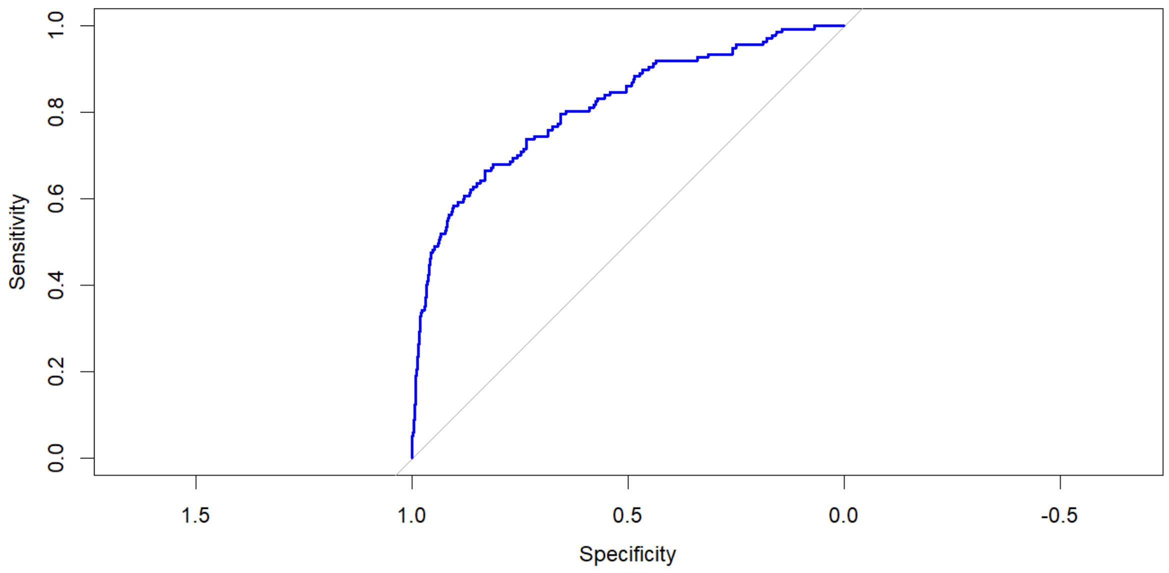

Table 2: Risk of not reaching the ODD 3.2.2 target for neonatal mortality (12 ‰ by 2030) by commune

Region	Commune	Risk of neonatal death
**BOUCLE DU MOUHOUN**	BAGASSI	0,354
**BOUCLE DU MOUHOUN**	BALAVÉ	0,459

**BOUCLE DU MOUHOUN**	BANA	0,416
**BOUCLE DU MOUHOUN**	BARANI	0,350
**BOUCLE DU MOUHOUN**	BOMBOROKUI	0,368
**BOUCLE DU MOUHOUN**	BONDOKUI	0,402
**BOUCLE DU MOUHOUN**	BOROMO	0,408
**BOUCLE DU MOUHOUN**	BOURASSO	0,411
**BOUCLE DU MOUHOUN**	DIIN	0,364
**BOUCLE DU MOUHOUN**	DJIBASSO	0,277
**BOUCLE DU MOUHOUN**	DOKUI	0,347
**BOUCLE DU MOUHOUN**	DOUMBALA	0,340
**BOUCLE DU MOUHOUN**	DOUROULA	0,279
**BOUCLE DU MOUHOUN**	DÉDOUGOU	0,337
**BOUCLE DU MOUHOUN**	FARA	0,500
**BOUCLE DU MOUHOUN**	GASSAN	0,230
**BOUCLE DU MOUHOUN**	GOMBORO	0,325
**BOUCLE DU MOUHOUN**	GOSSINA	0,347
**BOUCLE DU MOUHOUN**	KASSOUM	0,342
**BOUCLE DU MOUHOUN**	KIEMBARA	0,313
**BOUCLE DU MOUHOUN**	KOMBORI	0,370
**BOUCLE DU MOUHOUN**	KONA	0,427
**BOUCLE DU MOUHOUN**	KOUGNY	0,343
**BOUCLE DU MOUHOUN**	KOUKA	0,752
**BOUCLE DU MOUHOUN**	LANFIÈRA	0,349

**BOUCLE DU MOUHOUN**	LANKOUÉ	0,393
**BOUCLE DU MOUHOUN**	MADOUBA	0,383
**BOUCLE DU MOUHOUN**	NOUNA	0,396
**BOUCLE DU MOUHOUN**	OUARKOYE	0,538
**BOUCLE DU MOUHOUN**	OURI	0,459
**BOUCLE DU MOUHOUN**	POMPOÏ	0,344
**BOUCLE DU MOUHOUN**	POURA	0,525
**BOUCLE DU MOUHOUN**	PÂ	0,443
**BOUCLE DU MOUHOUN**	SAFANÉ	0,324
**BOUCLE DU MOUHOUN**	SAMI	0,548
**BOUCLE DU MOUHOUN**	SANABA	0,539
**BOUCLE DU MOUHOUN**	SIBY	0,526
**BOUCLE DU MOUHOUN**	SOLENZO	0,852
**BOUCLE DU MOUHOUN**	SÔNÔ	0,363
**BOUCLE DU MOUHOUN**	TANSILA	0,428
**BOUCLE DU MOUHOUN**	TCHÉRIBA	0,312
**BOUCLE DU MOUHOUN**	TOMA	0,328
**BOUCLE DU MOUHOUN**	TOUGAN	0,331
**BOUCLE DU MOUHOUN**	TOÉNI	0,332
**BOUCLE DU MOUHOUN**	YABA	0,292
**BOUCLE DU MOUHOUN**	YAHO	0,446
**BOUCLE DU MOUHOUN**	YÉ	0,275
**CASCADES**	BANFORA	0,847
**CASCADES**	BÉRÉGADOUGOU	0,589

**CASCADES**	DAKÔRÔ	0,684
**CASCADES**	DOUNA	0,619
**CASCADES**	KANKALABA	0,968
**CASCADES**	LOUMANA	0,750
**CASCADES**	MANGODARA	0,481
**CASCADES**	MOUSSODOUGOU	0,660
**CASCADES**	NIANGOLOKO	0,428
**CASCADES**	NIANKÔRÔDOUGOU	0,445
**CASCADES**	OUÉLÉNI	0,778
**CASCADES**	OUÔ	0,472
**CASCADES**	SIDÉRADOUGOU	0,325
**CASCADES**	SINDOU	0,666
**CASCADES**	SOUBAKANIÉDOUGOU	0,580
**CASCADES**	TIÉFORA	0,614
**CASCADES**	WOLONKOTO	0,606
**CENTRE**	KOMKI-IPALA	0,359
**CENTRE**	KOMSILGA	0,212
**CENTRE**	KOUBRI	0,522
**CENTRE**	OUAGADOUGOU	0,194
**CENTRE**	PABRÉ	0,511
**CENTRE**	SAABA	0,483
**CENTRE**	TANGUEN-DASSOURI	0,420
**CENTRE-EST**	ANDEMTENGA	0,438
**CENTRE-EST**	BAGRÉ	0,602
**CENTRE-EST**	BANÉ	0,602
**CENTRE-EST**	BASKOURÉ	0,435
**CENTRE-EST**	BISSIGA	0,630
**CENTRE-EST**	BITOU	0,563
**CENTRE-EST**	BOUSSOUMA	0,879
**CENTRE-EST**	BÉGUÉDO	0,683
**CENTRE-EST**	DIALGAYE	0,832
**CENTRE-EST**	DOURTENGA	0,665
**CENTRE-EST**	GARANGO	0,661
**CENTRE-EST**	GOUNGUEN	0,493
**CENTRE-EST**	KANDO	0,561
**CENTRE-EST**	KOMIN-YANGA	0,714
**CENTRE-EST**	KOMTOÈGA	0,655
**CENTRE-EST**	KOUPÈLA	0,739
**CENTRE-EST**	LALGAYE	0,740
**CENTRE-EST**	NIAOGHO	0,701

**CENTRE-EST**	OUARGAYE	0,744
**CENTRE-EST**	POUYTENGA	0,544
**CENTRE-EST**	SANGHA	0,823
**CENTRE-EST**	SOUDOUGUI	0,647
**CENTRE-EST**	TENKODOGO	0,945
**CENTRE-EST**	TENSOBENTENGA	0,591
**CENTRE-EST**	YARGATENGA	0,783
**CENTRE-EST**	YARGO	0,576
**CENTRE-EST**	YONDÉ	0,676
**CENTRE-EST**	ZABRÉ	0,361
**CENTRE-EST**	ZOAGA	0,363
**CENTRE-EST**	ZONSÉ	0,505
**CENTRE-NORD**	BARSALOGHO	0,411
**CENTRE-NORD**	BOALA	0,661
**CENTRE-NORD**	BOULSA	0,650
**CENTRE-NORD**	BOUROUM	0,462
**CENTRE-NORD**	BOURZANGA	0,528
**CENTRE-NORD**	BOUSSOUMA	0,390
**CENTRE-NORD**	DABLO	0,395
**CENTRE-NORD**	DARGO	0,502
**CENTRE-NORD**	GUIBARÉ	0,525
**CENTRE-NORD**	KAYA	0,225
**CENTRE-NORD**	KONGOUSSI	0,363
**CENTRE-NORD**	KORSIMORO	0,699
**CENTRE-NORD**	MANÉ	0,424
**CENTRE-NORD**	NAGBINGOU	0,478
**CENTRE-NORD**	NAMISSIGUIMA	0,389
**CENTRE-NORD**	NASSÉRÉ	0,387
**CENTRE-NORD**	PENSA	0,448
**CENTRE-NORD**	PIBAORÉ	0,743
**CENTRE-NORD**	PISSILA	0,486
**CENTRE-NORD**	ROLLO	0,286
**CENTRE-NORD**	ROUKÔ	0,344
**CENTRE-NORD**	SABSÉ	0,377
**CENTRE-NORD**	TIKARÉ	0,304
**CENTRE-NORD**	TOUGOURI	0,538
**CENTRE-NORD**	YALGO	0,474
**CENTRE-NORD**	ZIGA	0,573
**CENTRE-NORD**	ZIMTANGA	0,393
**CENTRE-NORD**	ZÉGUÉDÉGUEN	0,546
**CENTRE-OUEST**	BAKATA	0,360
**CENTRE-OUEST**	BIEHA	0,389
**CENTRE-OUEST**	BINGO	0,369

**CENTRE-OUEST**	BOUGNOUNOU	0,297
**CENTRE-OUEST**	BOURA	0,381
**CENTRE-OUEST**	DALÔ	0,369
**CENTRE-OUEST**	DASSA	0,284
**CENTRE-OUEST**	DIDYR	0,561
**CENTRE-OUEST**	GAÔ	0,376
**CENTRE-OUEST**	GODYR	0,409
**CENTRE-OUEST**	IMASGHO	0,314
**CENTRE-OUEST**	KASSOU	0,299
**CENTRE-OUEST**	KINDI	0,417
**CENTRE-OUEST**	KOKOLOGO	0,330
**CENTRE-OUEST**	KORDIÉ	0,458
**CENTRE-OUEST**	KOUDOUGOU	0,150
**CENTRE-OUEST**	KYON	0,257
**CENTRE-OUEST**	LÉO	0,555
**CENTRE-OUEST**	NANDIALA	0,333
**CENTRE-OUEST**	NANORO	0,529
**CENTRE-OUEST**	NIABOURI	0,395
**CENTRE-OUEST**	NÉBIÉLIANAYOU	0,363
**CENTRE-OUEST**	PELLA	0,397
**CENTRE-OUEST**	POA	0,291
**CENTRE-OUEST**	POUNI	0,435
**CENTRE-OUEST**	RAMONGO	0,305
**CENTRE-OUEST**	RÉO	0,233
**CENTRE-OUEST**	SABOU	0,293
**CENTRE-OUEST**	SAPOUY	0,272
**CENTRE-OUEST**	SIGLÉ	0,372
**CENTRE-OUEST**	SILLY	0,379
**CENTRE-OUEST**	SOA	0,353
**CENTRE-OUEST**	SOURGOU	0,249
**CENTRE-OUEST**	THIOU	0,349
**CENTRE-OUEST**	TO	0,347
**CENTRE-OUEST**	TÉNADO	0,230
**CENTRE-OUEST**	ZAMO	0,378
**CENTRE-OUEST**	ZAWARA	0,385
**CENTRE-SUD**	BINDÉ	0,906
**CENTRE-SUD**	BÉRÉ	0,608
**CENTRE-SUD**	DOULOUGOU	0,426
**CENTRE-SUD**	GAONGO	0,641
**CENTRE-SUD**	GOGO	0,798
**CENTRE-SUD**	GOMBOUSSOUGOU	0,572
**CENTRE-SUD**	GUIARO	0,400

**CENTRE-SUD**	GUIBA	0,589
**CENTRE-SUD**	IPELSÉ	0,419
**CENTRE-SUD**	KAYAO	0,287
**CENTRE-SUD**	KOMBISSIRI	0,690
**CENTRE-SUD**	MANGA	0,673
**CENTRE-SUD**	NOBÉRÉ	0,432
**CENTRE-SUD**	PO	0,565
**CENTRE-SUD**	SAPONÉ	0,338
**CENTRE-SUD**	TIÉBÉLÉ	0,410
**CENTRE-SUD**	TOÉCÉ	0,505
**CENTRE-SUD**	ZENKÔ	0,431
**CENTRE-SUD**	ZIOU	0,409
**EST**	BARTIÉBOUGOU	0,513
**EST**	BILANGA	0,459
**EST**	BOGANDÉ	0,432
**EST**	BOTOU	0,534
**EST**	DIABO	0,547
**EST**	DIAPAGA	0,543
**EST**	DIAPANGOU	0,511
**EST**	FADA-NGOURMA	0,698
**EST**	FOUTOURI	0,516
**EST**	GAYÉRI	0,516
**EST**	KANTCHARI	0,541
**EST**	KOALA	0,475
**EST**	KOMPIENGA	0,588
**EST**	LIPTOUGOU	0,488
**EST**	LÔGBOU	0,557
**EST**	MADJOARI	0,554
**EST**	MANI	0,336
**EST**	MATIAKOALI	0,561
**EST**	NAMOUNO	0,538
**EST**	PAMA	0,585
**EST**	PARTIAGA	0,545
**EST**	PIÉLA	0,718
**EST**	TAMBAGA	0,554
**EST**	TANSARGA	0,545
**EST**	THION	0,599
**EST**	TIBGA	0,407
**EST**	YAMBA	0,536
**HAUTS-BASSINS**	BAMA	0,575
**HAUTS-BASSINS**	BANZON	0,619
**HAUTS-BASSINS**	BOBO-DIOULASSO	0,538
**HAUTS-BASSINS**	BONI	0,458

**HAUTS-BASSINS**	BÉKUI	0,480
**HAUTS-BASSINS**	BÉRÉBA	0,475
**HAUTS-BASSINS**	DANDÉ	0,574
**HAUTS-BASSINS**	DJIGOUÈRA	0,672
**HAUTS-BASSINS**	FARAMANA	0,585
**HAUTS-BASSINS**	FO	0,592
**HAUTS-BASSINS**	FOUNZAN	0,450
**HAUTS-BASSINS**	HOUNDÉ	0,460
**HAUTS-BASSINS**	KANGALA	0,725
**HAUTS-BASSINS**	KARANGASSO-SAMBLA	0,616
**HAUTS-BASSINS**	KARANGASSO-VIGUÉ	0,490
**HAUTS-BASSINS**	KAYAN	0,595
**HAUTS-BASSINS**	KOTI	0,449
**HAUTS-BASSINS**	KOUMBIA	0,467
**HAUTS-BASSINS**	KOUNDOUGOU	0,595
**HAUTS-BASSINS**	KOURINION	0,641
**HAUTS-BASSINS**	KOUROUMA	0,603
**HAUTS-BASSINS**	KÔLÔKÔ	0,681
**HAUTS-BASSINS**	LÈNA	0,491
**HAUTS-BASSINS**	MORLABA	0,610
**HAUTS-BASSINS**	NDÔRÔLA	0,605
**HAUTS-BASSINS**	ORODARA	0,655
**HAUTS-BASSINS**	PADÉMA	0,566
**HAUTS-BASSINS**	PÉNI	0,548
**HAUTS-BASSINS**	SAMÔGÔGOUAN	0,640
**HAUTS-BASSINS**	SAMÔGÔYIRI	0,709
**HAUTS-BASSINS**	SATIRI	0,512
**HAUTS-BASSINS**	SINDO	0,627
**HAUTS-BASSINS**	TOUSSIANA	0,609
**NORD**	ARBOLLÉ	0,345
**NORD**	BAGARÉ	0,399
**NORD**	BAHN	0,303
**NORD**	BARGA	0,291
**NORD**	BASSI	0,584
**NORD**	BOKEN	0,498
**NORD**	BOUSSOU	0,461
**NORD**	GOMPONSOM	0,494
**NORD**	GOURSI	0,293
**NORD**	KALSAKA	0,405
**NORD**	KAÏN	0,339
**NORD**	KIRSI	0,431

**NORD**	KOSSOUKA	0,312
**NORD**	KOUMBRI	0,290
**NORD**	LA-TODEN	0,530
**NORD**	LÈBA	0,336
**NORD**	NAMISSIGUIMA	0,236
**NORD**	OUAHIGOUYA	0,139
**NORD**	OUINDIGUI	0,330
**NORD**	OULA	0,230
**NORD**	PILIMPIKOU	0,384
**NORD**	RAMBO	0,304
**NORD**	SAMBA	0,385
**NORD**	SENGUÈNÈGA	0,277
**NORD**	SOLLÉ	0,321
**NORD**	TANGAYE	0,314
**NORD**	THIOU	0,298
**NORD**	TITAO	0,225
**NORD**	TOUGO	0,396
**NORD**	YAKO	0,686
**NORD**	ZOGORÉ	0,320
**PLATEAU-CENTRAL**	AMBSOUYA	0,877
**PLATEAU-CENTRAL**	BOUDRI	0,643
**PLATEAU-CENTRAL**	BOUSSÉ	0,376
**PLATEAU-CENTRAL**	DAPEOLGO	0,588
**PLATEAU-CENTRAL**	KOGHO	0,714
**PLATEAU-CENTRAL**	LAYE	0,354
**PLATEAU-CENTRAL**	LOUMBILA	0,560
**PLATEAU-CENTRAL**	MÉGUÉ	0,679
**PLATEAU-CENTRAL**	MÔGTÉDO	0,534
**PLATEAU-CENTRAL**	NAGRÉONGO	0,601
**PLATEAU-CENTRAL**	NIOU	0,390
**PLATEAU-CENTRAL**	OURGOU-MANÉGA	0,535
**PLATEAU-CENTRAL**	SAOLGO	0,732

**PLATEAU-CENTRAL**	SOURGOUBILA	0,358
**PLATEAU-CENTRAL**	TOÈGUEN	0,422
**PLATEAU-CENTRAL**	ZAM	0,686
**PLATEAU-CENTRAL**	ZINIARÉ	0,860
**PLATEAU-CENTRAL**	ZITENGA	0,619
**PLATEAU-CENTRAL**	ZOUNGOU	0,854
**PLATEAU-CENTRAL**	ZÔRGHO	0,895
**SAHEL**	ARBINDA	0,414
**SAHEL**	BANI	0,444
**SAHEL**	BARABOULÉ	0,302
**SAHEL**	BOUNDORÉ	0,486
**SAHEL**	DIGUEL	0,326
**SAHEL**	DJIBO	0,142
**SAHEL**	DORI	0,531
**SAHEL**	DÉOU	0,427
**SAHEL**	FALAGOUNTOU	0,462
**SAHEL**	GORGADJI	0,454
**SAHEL**	GOROM-GOROM	0,442
**SAHEL**	KELBO	0,404
**SAHEL**	KOUTOUGOU	0,392
**SAHEL**	MANSILA	0,494
**SAHEL**	MARKOYE	0,453
**SAHEL**	NASSOUMBOU	0,344
**SAHEL**	OURSI	0,444
**SAHEL**	POBÉ-MENGAO	0,316
**SAHEL**	SAMPELGA	0,474
**SAHEL**	SEBBA	0,483
**SAHEL**	SEYTENGA	0,473
**SAHEL**	SOLHAN	0,483
**SAHEL**	TANKOUGOUNADIÉ	0,482
**SAHEL**	TIN-AKOFF	0,443
**SAHEL**	TITABÈ	0,474
**SAHEL**	TONGOMAYEL	0,364
**SUD-OUEST**	BATIÉ	0,489
**SUD-OUEST**	BOUROUM-BOUROUM	0,477
**SUD-OUEST**	BOUSSOU-KOULA	0,487

**SUD-OUEST**	BOUSSÉRA	0,484
**SUD-OUEST**	DANO	0,454
**SUD-OUEST**	DISSIHN	0,474
**SUD-OUEST**	DIÉBOUGOU	0,469
**SUD-OUEST**	DJIGOUÈ	0,477
**SUD-OUEST**	DOLO	0,472
**SUD-OUEST**	GAOUA	0,481
**SUD-OUEST**	GBOMBLORA	0,512
**SUD-OUEST**	GBONDJIGUI	0,471
**SUD-OUEST**	GUÉGUÉRÉ	0,461
**SUD-OUEST**	IÔLÔNIÔRÔ	0,472
**SUD-OUEST**	KAMPTI	0,479
**SUD-OUEST**	KOPER	0,442
**SUD-OUEST**	KPUÉRÉ	0,488
**SUD-OUEST**	LEGMOIN	0,492
**SUD-OUEST**	LOROPÉNI	0,475
**SUD-OUEST**	MALBA	0,479
**SUD-OUEST**	MIDEBDO	0,485
**SUD-OUEST**	NAKO	0,477
**SUD-OUEST**	NIÉGO	0,424
**SUD-OUEST**	ORONKUA	0,456
**SUD-OUEST**	OUÉSSA	0,425
**SUD-OUEST**	PÉRIGBAN	0,480
**SUD-OUEST**	TIANKOURA	0,472
**SUD-OUEST**	ZAMBO	0,470

**Région**	Commune	Risk probality(threshold 0.012)
**BOUCLE DU MOUHOUN**	BAGASSI	0,165
**BOUCLE DU MOUHOUN**	BALAVÉ	0,186
**BOUCLE DU MOUHOUN**	BANA	0,169
**BOUCLE DU MOUHOUN**	BARANI	0,179
**BOUCLE DU MOUHOUN**	BOMBOROKUI	0,190
**BOUCLE DU MOUHOUN**	BONDOKUI	0,165
**BOUCLE DU MOUHOUN**	BOROMO	0,160
**BOUCLE DU MOUHOUN**	BOURASSO	0,177
**BOUCLE DU MOUHOUN**	DIIN	0,189
**BOUCLE DU MOUHOUN**	DJIBASSO	0,193
**BOUCLE DU MOUHOUN**	DOKUI	0,175
**BOUCLE DU MOUHOUN**	DOUMBALA	0,185
**BOUCLE DU MOUHOUN**	DOUROULA	0,168
**BOUCLE DU MOUHOUN**	DÉDOUGOU	0,163
**BOUCLE DU MOUHOUN**	FARA	0,162

**BOUCLE DU MOUHOUN**	GASSAN	0,159
**BOUCLE DU MOUHOUN**	GOMBORO	0,176
**BOUCLE DU MOUHOUN**	GOSSINA	0,169
**BOUCLE DU MOUHOUN**	KASSOUM	0,172
**BOUCLE DU MOUHOUN**	KIEMBARA	0,166
**BOUCLE DU MOUHOUN**	KOMBORI	0,207
**BOUCLE DU MOUHOUN**	KONA	0,172
**BOUCLE DU MOUHOUN**	KOUGNY	0,176
**BOUCLE DU MOUHOUN**	KOUKA	0,180
**BOUCLE DU MOUHOUN**	LANFIÈRA	0,177
**BOUCLE DU MOUHOUN**	LANKOUÉ	0,183
**BOUCLE DU MOUHOUN**	MADOUBA	0,231
**BOUCLE DU MOUHOUN**	NOUNA	0,178
**BOUCLE DU MOUHOUN**	OUARKOYE	0,174
**BOUCLE DU MOUHOUN**	OURI	0,162
**BOUCLE DU MOUHOUN**	POMPOÏ	0,176
**BOUCLE DU MOUHOUN**	POURA	0,174
**BOUCLE DU MOUHOUN**	PÂ	0,173
**BOUCLE DU MOUHOUN**	SAFANÉ	0,163
**BOUCLE DU MOUHOUN**	SAMI	0,183
**BOUCLE DU MOUHOUN**	SANABA	0,176
**BOUCLE DU MOUHOUN**	SIBY	0,174
**BOUCLE DU MOUHOUN**	SOLENZO	0,170
**BOUCLE DU MOUHOUN**	SÔNÔ	0,172
**BOUCLE DU MOUHOUN**	TANSILA	0,187
**BOUCLE DU MOUHOUN**	TCHÉRIBA	0,157
**BOUCLE DU MOUHOUN**	TOMA	0,171
**BOUCLE DU MOUHOUN**	TOUGAN	0,162
**BOUCLE DU MOUHOUN**	TOÉNI	0,172
**BOUCLE DU MOUHOUN**	YABA	0,163
**BOUCLE DU MOUHOUN**	YAHO	0,174
**BOUCLE DU MOUHOUN**	YÉ	0,166
**CASCADES**	BANFORA	0,192
**CASCADES**	BÉRÉGADOUGOU	0,196
**CASCADES**	DAKÔRÔ	0,206
**CASCADES**	DOUNA	0,207
**CASCADES**	KANKALABA	0,200
**CASCADES**	LOUMANA	0,203
**CASCADES**	MANGODARA	0,197
**CASCADES**	MOUSSODOUGOU	0,190
**CASCADES**	NIANGOLOKO	0,191
**CASCADES**	NIANKÔRÔDOUGOU	0,219

**CASCADES**	OUÉLÉNI	0,202
**CASCADES**	OUÔ	0,181
**CASCADES**	SIDÉRADOUGOU	0,183
**CASCADES**	SINDOU	0,194
**CASCADES**	SOUBAKANIÉDOU GOU	0,196
**CASCADES**	TIÉFORA	0,188
**CASCADES**	WOLONKOTO	0,198
**CENTRE**	KOMKI-IPALA	0,175
**CENTRE**	KOMSILGA	0,163
**CENTRE**	KOUBRI	0,157
**CENTRE**	OUAGADOUGOU	0,162
**CENTRE**	PABRÉ	0,161
**CENTRE**	SAABA	0,167
**CENTRE**	TANGUEN-DASSOURI	0,163
**CENTRE-EST**	ANDEMTENGA	0,165
**CENTRE-EST**	BAGRÉ	0,174
**CENTRE-EST**	BANÉ	0,183
**CENTRE-EST**	BASKOURÉ	0,180
**CENTRE-EST**	BISSIGA	0,174
**CENTRE-EST**	BITOU	0,176
**CENTRE-EST**	BOUSSOUMA	0,176
**CENTRE-EST**	BÉGUÉDO	0,182
**CENTRE-EST**	DIALGAYE	0,173
**CENTRE-EST**	DOURTENGA	0,188
**CENTRE-EST**	GARANGO	0,163
**CENTRE-EST**	GOUNGUEN	0,169
**CENTRE-EST**	KANDO	0,166
**CENTRE-EST**	KOMIN-YANGA	0,177
**CENTRE-EST**	KOMTOÈGA	0,175
**CENTRE-EST**	KOUPÈLA	0,164
**CENTRE-EST**	LALGAYE	0,175
**CENTRE-EST**	NIAOGHO	0,174
**CENTRE-EST**	OUARGAYE	0,183
**CENTRE-EST**	POUYTENGA	0,177
**CENTRE-EST**	SANGHA	0,191
**CENTRE-EST**	SOUDOUGUI	0,183
**CENTRE-EST**	TENKODOGO	0,169
**CENTRE-EST**	TENSOBENTENGA	0,172
**CENTRE-EST**	YARGATENGA	0,195
**CENTRE-EST**	YARGO	0,181
**CENTRE-EST**	YONDÉ	0,186

**CENTRE-EST**	ZABRÉ	0,183
**CENTRE-EST**	ZOAGA	0,207
**CENTRE-EST**	ZONSÉ	0,184
**CENTRE-NORD**	BARSALOGHO	0,168
**CENTRE-NORD**	BOALA	0,172
**CENTRE-NORD**	BOULSA	0,166
**CENTRE-NORD**	BOUROUM	0,171
**CENTRE-NORD**	BOURZANGA	0,165
**CENTRE-NORD**	BOUSSOUMA	0,164
**CENTRE-NORD**	DABLO	0,176
**CENTRE-NORD**	DARGO	0,172
**CENTRE-NORD**	GUIBARÉ	0,170
**CENTRE-NORD**	KAYA	0,163
**CENTRE-NORD**	KONGOUSSI	0,167
**CENTRE-NORD**	KORSIMORO	0,164
**CENTRE-NORD**	MANÉ	0,159
**CENTRE-NORD**	NAGBINGOU	0,183
**CENTRE-NORD**	NAMISSIGUIMA	0,171
**CENTRE-NORD**	NASSÉRÉ	0,174
**CENTRE-NORD**	PENSA	0,179
**CENTRE-NORD**	PIBAORÉ	0,167
**CENTRE-NORD**	PISSILA	0,163
**CENTRE-NORD**	ROLLO	0,172
**CENTRE-NORD**	ROUKÔ	0,173
**CENTRE-NORD**	SABSÉ	0,168
**CENTRE-NORD**	TIKARÉ	0,169
**CENTRE-NORD**	TOUGOURI	0,167
**CENTRE-NORD**	YALGO	0,179
**CENTRE-NORD**	ZIGA	0,166
**CENTRE-NORD**	ZIMTANGA	0,180
**CENTRE-NORD**	ZÉGUÉDÉGUEN	0,172
**CENTRE-OUEST**	BAKATA	0,168
**CENTRE-OUEST**	BIEHA	0,166
**CENTRE-OUEST**	BINGO	0,175
**CENTRE-OUEST**	BOUGNOUNOU	0,166
**CENTRE-OUEST**	BOURA	0,179
**CENTRE-OUEST**	DALÔ	0,177
**CENTRE-OUEST**	DASSA	0,165
**CENTRE-OUEST**	DIDYR	0,159
**CENTRE-OUEST**	GAÔ	0,171
**CENTRE-OUEST**	GODYR	0,170
**CENTRE-OUEST**	IMASGHO	0,175
**CENTRE-OUEST**	KASSOU	0,163

**CENTRE-OUEST**	KINDI	0,167
**CENTRE-OUEST**	KOKOLOGO	0,166
**CENTRE-OUEST**	KORDIÉ	0,164
**CENTRE-OUEST**	KOUDOUGOU	0,165
**CENTRE-OUEST**	KYON	0,182
**CENTRE-OUEST**	LÉO	0,185
**CENTRE-OUEST**	NANDIALA	0,170
**CENTRE-OUEST**	NANORO	0,156
**CENTRE-OUEST**	NIABOURI	0,169
**CENTRE-OUEST**	NÉBIÉLIANAYOU	0,166
**CENTRE-OUEST**	PELLA	0,175
**CENTRE-OUEST**	POA	0,163
**CENTRE-OUEST**	POUNI	0,156
**CENTRE-OUEST**	RAMONGO	0,165
**CENTRE-OUEST**	RÉO	0,162
**CENTRE-OUEST**	SABOU	0,160
**CENTRE-OUEST**	SAPOUY	0,158
**CENTRE-OUEST**	SIGLÉ	0,166
**CENTRE-OUEST**	SILLY	0,159
**CENTRE-OUEST**	SOA	0,164
**CENTRE-OUEST**	SOURGOU	0,172
**CENTRE-OUEST**	THIOU	0,168
**CENTRE-OUEST**	TO	0,165
**CENTRE-OUEST**	TÉNADO	0,156
**CENTRE-OUEST**	ZAMO	0,162
**CENTRE-OUEST**	ZAWARA	0,162
**CENTRE-SUD**	BINDÉ	0,171
**CENTRE-SUD**	BÉRÉ	0,170
**CENTRE-SUD**	DOULOUGOU	0,167
**CENTRE-SUD**	GAONGO	0,166
**CENTRE-SUD**	GOGO	0,166
**CENTRE-SUD**	GOMBOUSSOUGOU	0,167
**CENTRE-SUD**	GUIARO	0,176
**CENTRE-SUD**	GUIBA	0,174
**CENTRE-SUD**	IPELSÉ	0,183
**CENTRE-SUD**	KAYAO	0,159
**CENTRE-SUD**	KOMBISSIRI	0,175
**CENTRE-SUD**	MANGA	0,180
**CENTRE-SUD**	NOBÉRÉ	0,168
**CENTRE-SUD**	PO	0,168
**CENTRE-SUD**	SAPONÉ	0,162
**CENTRE-SUD**	TIÉBÉLÉ	0,187

**CENTRE-SUD**	TOÉCÉ	0,165
**CENTRE-SUD**	ZENKÔ	0,208
**CENTRE-SUD**	ZIOU	0,188
**EST**	BARTIÉBOUGOU	0,183
**EST**	BILANGA	0,169
**EST**	BOGANDÉ	0,169
**EST**	BOTOU	0,218
**EST**	DIABO	0,177
**EST**	DIAPAGA	0,201
**EST**	DIAPANGOU	0,186
**EST**	FADA-NGOURMA	0,176
**EST**	FOUTOURI	0,193
**EST**	GAYÉRI	0,175
**EST**	KANTCHARI	0,202
**EST**	KOALA	0,174
**EST**	KOMPIENGA	0,206
**EST**	LIPTOUGOU	0,175
**EST**	LÔGBOU	0,194
**EST**	MADJOARI	0,210
**EST**	MANI	0,175
**EST**	MATIAKOALI	0,177
**EST**	NAMOUNO	0,213
**EST**	PAMA	0,185
**EST**	PARTIAGA	0,197
**EST**	PIÉLA	0,187
**EST**	TAMBAGA	0,192
**EST**	TANSARGA	0,205
**EST**	THION	0,181
**EST**	TIBGA	0,179
**EST**	YAMBA	0,177
**HAUTS-BASSINS**	BAMA	0,177
**HAUTS-BASSINS**	BANZON	0,198
**HAUTS-BASSINS**	BOBO-DIOULASSO	0,176
**HAUTS-BASSINS**	BONI	0,179
**HAUTS-BASSINS**	BÉKUI	0,176
**HAUTS-BASSINS**	BÉRÉBA	0,181
**HAUTS-BASSINS**	DANDÉ	0,194
**HAUTS-BASSINS**	DJIGOUÈRA	0,188
**HAUTS-BASSINS**	FARAMANA	0,190
**HAUTS-BASSINS**	FO	0,191
**HAUTS-BASSINS**	FOUNZAN	0,169
**HAUTS-BASSINS**	HOUNDÉ	0,166
**HAUTS-BASSINS**	KANGALA	0,198

**HAUTS-BASSINS**	KARANGASSO-SAMBLA	0,179
**HAUTS-BASSINS**	KARANGASSO-VIGUÉ	0,174
**HAUTS-BASSINS**	KAYAN	0,188
**HAUTS-BASSINS**	KOTI	0,174
**HAUTS-BASSINS**	KOUMBIA	0,170
**HAUTS-BASSINS**	KOUNDOUGOU	0,180
**HAUTS-BASSINS**	KOURINION	0,192
**HAUTS-BASSINS**	KOUROUMA	0,182
**HAUTS-BASSINS**	KÔLÔKÔ	0,196
**HAUTS-BASSINS**	LÈNA	0,173
**HAUTS-BASSINS**	MORLABA	0,204
**HAUTS-BASSINS**	NDÔRÔLA	0,194
**HAUTS-BASSINS**	ORODARA	0,198
**HAUTS-BASSINS**	PADÉMA	0,175
**HAUTS-BASSINS**	PÉNI	0,182
**HAUTS-BASSINS**	SAMÔGÔGOUAN	0,187
**HAUTS-BASSINS**	SAMÔGÔYIRI	0,194
**HAUTS-BASSINS**	SATIRI	0,171
**HAUTS-BASSINS**	SINDO	0,204
**HAUTS-BASSINS**	TOUSSIANA	0,188
**NORD**	ARBOLLÉ	0,160
**NORD**	BAGARÉ	0,170
**NORD**	BAHN	0,181
**NORD**	BARGA	0,182
**NORD**	BASSI	0,173
**NORD**	BOKEN	0,155
**NORD**	BOUSSOU	0,164
**NORD**	GOMPONSOM	0,166
**NORD**	GOURSI	0,158
**NORD**	KALSAKA	0,161
**NORD**	KAÏN	0,202
**NORD**	KIRSI	0,174
**NORD**	KOSSOUKA	0,176
**NORD**	KOUMBRI	0,179
**NORD**	LA-TODEN	0,170
**NORD**	LÈBA	0,175
**NORD**	NAMISSIGUIMA	0,176
**NORD**	OUAHIGOUYA	0,169
**NORD**	OUINDIGUI	0,174
**NORD**	OULA	0,175
**NORD**	PILIMPIKOU	0,172

**NORD**	RAMBO	0,167
**NORD**	SAMBA	0,161
**NORD**	SENGUÈNÈGA	0,160
**NORD**	SOLLÉ	0,190
**NORD**	TANGAYE	0,182
**NORD**	THIOU	0,173
**NORD**	TITAO	0,168
**NORD**	TOUGO	0,171
**NORD**	YAKO	0,161
**NORD**	ZOGORÉ	0,172
**PLATEAU-CENTRAL**	AMBSOUYA	0,168
**PLATEAU-CENTRAL**	BOUDRI	0,159
**PLATEAU-CENTRAL**	BOUSSÉ	0,162
**PLATEAU-CENTRAL**	DAPEOLGO	0,158
**PLATEAU-CENTRAL**	KOGHO	0,171
**PLATEAU-CENTRAL**	LAYE	0,175
**PLATEAU-CENTRAL**	LOUMBILA	0,167
**PLATEAU-CENTRAL**	MÉGUÉ	0,182
**PLATEAU-CENTRAL**	MÔGTÉDO	0,168
**PLATEAU-CENTRAL**	NAGRÉONGO	0,167
**PLATEAU-CENTRAL**	NIOU	0,163
**PLATEAU-CENTRAL**	OURGOU-MANÉGA	0,167
**PLATEAU-CENTRAL**	SAOLGO	0,164
**PLATEAU-CENTRAL**	SOURGOUBILA	0,163
**PLATEAU-CENTRAL**	TOÈGUEN	0,181
**PLATEAU-CENTRAL**	ZAM	0,159
**PLATEAU-CENTRAL**	ZINIARÉ	0,155
**PLATEAU-CENTRAL**	ZITENGA	0,173
**PLATEAU-CENTRAL**	ZOUNGOU	0,171
**PLATEAU-CENTRAL**	ZÔRGHO	0,162
**SAHEL**	ARBINDA	0,169
**SAHEL**	BANI	0,174
**SAHEL**	BARABOULÉ	0,180
**SAHEL**	BOUNDORÉ	0,201
**SAHEL**	DIGUEL	0,193
**SAHEL**	DJIBO	0,176
**SAHEL**	DORI	0,182
**SAHEL**	DÉOU	0,187
**SAHEL**	FALAGOUNTOU	0,192
**SAHEL**	GORGADJI	0,180
**SAHEL**	GOROM-GOROM	0,181
**SAHEL**	KELBO	0,181
**SAHEL**	KOUTOUGOU	0,184

**SAHEL**	MANSILA	0,183
**SAHEL**	MARKOYE	0,200
**SAHEL**	NASSOUMBOU	0,186
**SAHEL**	OURSI	0,200
**SAHEL**	POBÉ-MENGAO	0,176
**SAHEL**	SAMPELGA	0,189
**SAHEL**	SEBBA	0,190
**SAHEL**	SEYTENGA	0,191
**SAHEL**	SOLHAN	0,184
**SAHEL**	TANKOUGOUNADI É	0,200
**SAHEL**	TIN-AKOFF	0,196
**SAHEL**	TITABÈ	0,180
**SAHEL**	TONGOMAYEL	0,172
**SUD-OUEST**	BATIÉ	0,215
**SUD-OUEST**	BOUROUM-BOUROUM	0,196
**SUD-OUEST**	BOUSSOU-KOULA	0,224
**SUD-OUEST**	BOUSSÉRA	0,203
**SUD-OUEST**	DANO	0,173
**SUD-OUEST**	DISSIHN	0,182
**SUD-OUEST**	DIÉBOUGOU	0,185
**SUD-OUEST**	DJIGOUÈ	0,201
**SUD-OUEST**	DOLO	0,191
**SUD-OUEST**	GAOUA	0,195
**SUD-OUEST**	GBOMBLORA	0,207
**SUD-OUEST**	GBONDJIGUI	0,177
**SUD-OUEST**	GUÉGUÉRÉ	0,170
**SUD-OUEST**	IÔLÔNIÔRÔ	0,187
**SUD-OUEST**	KAMPTI	0,202
**SUD-OUEST**	KOPER	0,175
**SUD-OUEST**	KPUÉRÉ	0,243
**SUD-OUEST**	LEGMOIN	0,225
**SUD-OUEST**	LOROPÉNI	0,188
**SUD-OUEST**	MALBA	0,198
**SUD-OUEST**	MIDEBDO	0,204
**SUD-OUEST**	NAKO	0,193
**SUD-OUEST**	NIÉGO	0,192
**SUD-OUEST**	ORONKUA	0,181
**SUD-OUEST**	OUÉSSA	0,185
**SUD-OUEST**	PÉRIGBAN	0,203
**SUD-OUEST**	TIANKOURA	0,186
**SUD-OUEST**	ZAMBO	0,184
